# Minimally invasive oesophagectomy versus open esophagectomy for resectable esophageal cancer: a meta-analysis

**DOI:** 10.1186/s12957-016-1062-7

**Published:** 2016-12-08

**Authors:** Waresijiang Yibulayin, Sikandaer Abulizi, Hongbo Lv, Wei Sun

**Affiliations:** Department of Thoracic Surgery, Tumor Hospital of Xinjiang Medical University, Urumqi, China

**Keywords:** Minimally invasive esophagectomy, Open esophagectomy, Complications, Mortality

## Abstract

**Background:**

Open esophagectomy (OE) is associated with significant morbidity and mortality. Minimally invasive oesophagectomy (MIO) reduces complications in resectable esophageal cancer. The aim of this study is to explore the superiority of MIO in reducing complications and in-hospital mortality than OE.

**Methods:**

MEDLINE, Embase, Science Citation Index, Wanfang, and Wiley Online Library were thoroughly searched. Odds ratio (OR)/weighted mean difference (WMD) with a 95% confidence interval (CI) was used to assess the strength of association.

**Results:**

Fifty-seven studies containing 15,790 cases of resectable esophageal cancer were included. MIO had less intraoperative blood loss, short hospital stay, and high operative time (*P* < 0.05) than OE. MIO also had reduced incidence of total complications; (OR = 0.700, 95% CI = 0.626 ~ 0.781, *P*_*V*_ < 0.05), pulmonary complications (OR = 0.527, 95% CI = 0431 ~ 0.645, *P*_*V*_ < 0.05), cardiovascular complications (OR = 0.770, 95% CI = 0.681 ~ 0.872, *P*_*V*_ < 0.05), and surgical technology related (STR) complications (OR = 0.639, 95% CI = 0.522 ~ 0.781, *P*_*V*_ < 0.05), as well as lower in-hospital mortality (OR = 0.668, 95% CI = 0.539 ~ 0.827, *P*_*V*_ < 0.05). However, the number of harvested lymph nodes, intensive care unit (ICU) stay, gastrointestinal complications, anastomotic leak (AL), and recurrent laryngeal nerve palsy (RLNP) had no significant difference.

**Conclusions:**

MIO is superior to OE in terms of perioperative complications and in-hospital mortality.

## Background

A global incidence of esophageal cancer has increased by 50% in the past two decades. Each year, around 482,300 people are diagnosed with esophageal cancer, and 84.3% die of the disease worldwide [1, 2]. At present, the primary method of treating patients with esophageal cancer has been surgery. However, the traditional open esophagectomy (OE) procedure has high complication rates resulting in significant morbidity and mortality [3, 4]. Various studies showed in-hospital mortality between 1.2 and 8.8% [4–7], even as high as 29% [8].

Minimally invasive oesophagectomy (MIO), which was first described in the 1990s [9, 10], was attributed to be superior in reducing postoperative outcomes, without compromising oncological outcomes and avoiding thoracotomy and laparotomy. The basis of minimally invasive techniques in esophageal surgery is to maintain the therapy effectiveness and quality of traditional operations, while reducing perioperative injury. Nevertheless, the real benefits of minimally invasive approach for esophagectomy are still controversial [11–13]. A number of meta-analyses and even randomized controlled trials demonstrated MIO to be superior in reducing risk of postoperative outcomes, but their results are not very consistent, especially on the issue of in-hospital mortality [14–30]. Furthermore, these studies ignored preoperative clinical data and other Chinese relevant literatures. We, therefore, performed a meta-analysis combining the relevant publications and comprehensively assess the superiority of MIO.

## Materials and methods

### Search strategy

MEDLINE, Embase, Science Citation Index, Wanfang, and Wiley Online Library were thoroughly searched with terms “Minimally Invasive Esophagectomy” or “Open Esophagectomy,” “Esophagectomy,””MIE,” “laparasc,” “thoracosc,” “VATS,” “transhiatal” (date until May 2016). Relevant literatures containing full text were back tracked thoroughly, while abstracts and unpublished reports were excluded.

#### Selections of studies

##### Inclusion criteria

The inclusion criteria are as follows: (1) randomized or non-randomized controlled studies with parallel controls, (2) comparison on OE versus MIO, (3) sufficient data of estimated odds ratios (ORs)/weighted mean difference (WMD) and 95% confidence intervals (CIs).

##### Exclusion criteria

The exclusion criteria are as follows: (1) studies that were not compared or case report, (2) incomplete literature, and (3) overlapped studies.

#### Data extraction

Two investigators read all the included literatures carefully and extracted all the data, such as first author, published year, numbers of case and controls, outcomes of interest, etc. If two investigators have divergent ideas on any data, the third investigator would be asked to check and reach consensus on the data.

#### Outcomes of interest

1. Definition of MIO was thoracoscopic/laparotomy-assisted esophagectomy, hybrid minimally invasive esophagectomy, total thoracoscopic/hand-assisted thoracotomy, hand-assisted laparotomy, or minilaparotomy/laparoscopic esophagectomy.
2. Preoperative clinical data included age, neoadjuvant therapy, comorbidity, TNM staging, and gender.
3. Postoperative data contained operative duration, blood loss, intensive care unit (ICU) stay, hospital stay, and harvested lymph nodes.
4. The complications are as follows. (1) Mortality included in-hospital mortality and 30-day mortality. (2) Pulmonary complications included pneumonia, respiratory failure, adult respiratory distress syndrome (ARDS), etc. (3) Cardiovascular complications included arrhythmia, heart failure, acute myocardial infarction, deep vein thrombosis, pulmonary embolism, etc. (4) Gastrointestinal complications included gastric tip necrosis, anastomotic stricture, delayed gastric emptying, gastric volvulus, etc. (5) Surgical technology related (STR) complications included splenic laceration, tracheal laceration, pneumothorax, chylothorax, hemorrhage, etc.

#### Statistical analysis

Data was analyzed using STATA 11 (Stata Corp LP, College Station, Texas, 2011). Fixed or random effects models [31] were used. Odds ratio (OR) was used for categorical variables, while weighted mean difference (WMD) was used for continuous variables, such as operative time, harvested lymph nodes, and blood loss [32]. *Q* test was used to check the heterogeneity among each study. If the heterogeneity was high (*I* ^2^ > 50%), Random Effects Model was used to calculate the pooled OR/WMD. Otherwise, the fixed effects model was used [33]. If the heterogeneity test was statistically significant, sensitivity analysis, subgroup analysis, and Galbraith Plot Analysis were performed to find out potential origin of heterogeneity. Egger’s Test and Begg’s Funnel Plot were used for diagnosis of potential publication bias [34]. A *P* value <0.05 was considered as statistical significance. Duval and Tweedie nonparametric “trim and fill” procedure was used to assess the possible effect of publication bias [35].

The Newcastle Ottawa Quality Assessment Scale was used to assess the validity and quality of studies [36], as recommended in the Cochrane Handbook [37]. This scale assigns a star rating based on pre-specified criteria. A total number of quality star ranged from one (low quality) to nine (high quality). A maximum of one star can be attained for each category, except comparability, which has maximum of two stars. The more the stars, the higher is the quality of study.

## Results

### Study characteristics

**Fig. 1:**
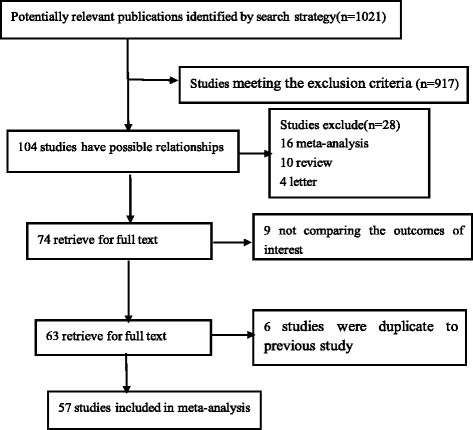
Study flow chart explaining the selection of 57 studies included in the meta-analysis

A flow chart of the literature search process is shown in Fig. 1. A total of 1021 unique records were identified by search strategy; 917 records were excluded; 16 studies were meta-analyses or systematic overviews [14–19]; ten were review; and four were letter; nine studies did not compare the outcomes of interest [3, 5–12], and six studies were duplicate to previous study. Therefore, 57 studies containing 15,790 cases (both MIO and OE) were included in this meta-analysis [30, 38–93].

Preoperative clinical data as well as quality star ranging from 6 to 8 are shown in Table 1. Of 15,790 cases, 5235 (33.2%) were MIO and 10,555 (66.8%) were OE. Thirty one studies were done in European countries and 26 in Asian countries, where 13 were from China [45–57]. Moreover, 39 studies involved total MIE, 12 studies thoracoscopic-assisted MIE (TA), and seven studies were hybrid (TA + MIE). TNM staging were reported in 40 studies (6265 cases), where 1973 patients (64.4%) in the MIO group and 1042 patients (32.5%) in the OE group were of early stage (stages I and II), mainly male (78.4% (MIO) vs 68.3% (OE)).

**Table 1:** Characteristics of included studies in this meta-analysis

Study	Year	Country	Cases	Gender (M)	Age, years	NT	NOS	Hybrid	Preoperative comorbidity (MIO/OE)			TNM stage (MIO/OE)	
			(MIO/OE)	(MIO/OE)	(MIO/OE)	(MIO/OE)			Cardiovascular	Pulmonary	Diabetes	0 + I + II	III + IV
Nguyen	2000	USA	18/36	7/29	64 ± 12/67 ± 8	9/9	6	MIE	NR	NR	NR	NR	NR
Osugi	2003	Japan	77/72	64/57	63.7 ± 9 · 6/64 · ±9 · 3	NR	7	TA	NR	NR	NR	NR	NR
Kunisaki	2004	Japan	15/30	12/21	62.3 ± 8.1/63 ± 6	NR	6	MIE	NR	NR	NR	NR	NR
Bernabe	2004	USA	17/14	16/11	63.9 ± 13.5/64.1 ± 10.7	NR	6	TA	NR	NR	NR	NR	NR
Van den Broek	2004	Netherlands	25/20	19/14	63 ± 8/64 ± 8	17/4	7	TA	NR	NR	NR	8/10	17/10
Braghetto	2006	Chile	47/119	NR	NR	0/0	8	MIE	NR	NR	NR	41/80	6/39
Bresadola	2006	Italy	14/14	8/13	61.9 ± 7.7/59.3 ± 10.9	NR	6	MIE	NR	NR	NR	11/6	3/8
Shiraishi	2006	Japan	116/37	94/31	61.5 ± 8.1/66.5 ± 9.3	26/10	7	Hybrid	NR	NR	NR	NR	NR
Smithers	2007	Australia	332/114	267/104	64 (27–85)/62.5 (29–81)	136/29	8	Hybrid	76/22	NR	27/4	192/36	118/75
Benzoni	2007	Italy	9/13	6/11	63.6 ± 2.6/60.2 ± 2.4	6/6	8	TA	NR	2/4	NR	9/7	0/6
Fabian	2008	USA	22/43	16/31	63 (46–86)/61 (35–82)	9/16	7	MIE	NR	NR	NR	14/25	7/19
Parameswaran	2009	UK	50/30	45/21	67 (47–81)/68 (47–81)	32/12	8	MIE	NR	NR	NR	27/17	23/13
Saha	2009	UK	16/28	13/24	65 (50–80)/64 (35–78)	NR	8	MIE	NR	NR	NR	NR	NR
Zingg	2009	Australia	56/98	45/71	66.3 ± 1.3/67.8 ± 1.1	40/48	8	MIE	4/7	13/35	6/12	35/47	21/42
Pham	2010	USA	44/46	41/33	63 ± 8.6/61 ± 10.7	29/23	6	MIE	NR	NR	NR	20/20	20/19
Perry	2010	USA	21/21	18/17	69 ± 8/61 ± 9	NR	7	MIE	NR	NR	NR	NR	NR
Hamouda	2010	UK	51/24	44/23	62/60	44/20	7	MIE	NR	NR	NR	NR	NR
Safranek	2010	UK	75/46	53/38	60 (44–77)/64(41–74)	71/34	7	Hybrid	NR	NR	NR	31/29	44/17
Schoppmann	2010	Australia	31/31	25/21	61.5 (36–75)/58.6 (34–77)	15/7	8	MIE	6/8	10/8	1/1	18/19	13/12
Schröder	2010	Germany	238/181	198/151	61.1 (60–62)/57.8 (56–59)	144/66	6	TA	NR	NR	NR	NR	NR
Mehran	2011	USA	44/44	43/40	61.0 (42–79)/62.5 (38–83)	31/30	7	MIE	NR	NR	NR	23/21	16/20
Berger	2011	USA	65/53	51/38	61 (41–78)/62 (40–86)	28/43	6	MIE	NR	NR	NR	52/41	13/12
Lee	2011	Japan	74/64	73/61	59.7 ± 10.3/56.6 ± 11.6	47/52	8	Hybrid	NR	NR	NR	54/49	20/15
Nafteux	2011	Belgium	65/101	52/81	63 (41–82)/64 (29–82)	NR	8	MIE	11/24	6/13	6/12	NR	NR
Yamasaki	2011	Japan	109/107	87/95	64.6 ± 8.5/64.7 ± 8.0	85/68	8	TA	20/20	11/13	10/6	NR	NR
Biere	2012	Netherlands	59/56	43/46	62 (34–75)/62 (42–75)	59/56	8	MIE	NR	NR	NR	31/26	15/19
Maas	2012	Netherlands	50/50	41/33	62.5 (57–69)/65 (57–69)	23/13	8	MIE	NR	NR	NR	19/19	31/31
Briez	2012	France	140/140	110/117	NR	67/69	8	TA	NR	NR	NR	92/89	48/51
Kinjo	2012	Japan	106/79	87/70	62.7 ± 7.4/63.3 ± 8.6	54/11	7	MIE	NR	NR	NR	65/45	41/34
Mamidanna	2012	UK	1155/6347	892/4870	NR	NR	7	MIE	400/2234	141/782	90/598	NR	NR
Sihag	2012	USA	38/76	29/61	61.4 ± 8.1/63.3 ± 9.3	25/46	7	MIE	6/16	8/13	NR	29/53	9/23
Sundaram	2012	USA	47/57	38/52	67.3 (42–79)/61.7 (34–84)	35/40	8	MIE	33/42	NR	11/14	NR	NR
Tsujimoto	2012	Japan	22/27	21/21	70 ± 5.4/67 ± 10.1	8/16	6	TA	NR	NR	NR	12/14	10/13
Javidfar	2012	USA	92/165	71/122	65 (56–74)/68 (60–74)	51/96	7	MIE	9/23	9/23	22/35	65/96	27/69
Bailey	2013	UK	39/31	32/27	65 (37–78)/62 (38–78)	33/31	7	TA	NR	NR	NR	NR	NR
Ichikawa	2013	Japan	152/163	129/145	63.8 ± 8.5/64.6 ± 8.6	54/64	8	TA	23/35	21/24	26/37	101/81	51/79
Kitagawa	2013	Japan	45/47	35/40	63 (47–77)/64 (39–83)	8/11	7	MIE	NR	NR	8/8	NR	NR
Noble	2013	UK	53/53	43/45	66 (45–85)/64 (36–81)	13/11	8	MIE	NR	NR	NR	NR	NR
Parameswaran	2013	UK	67/19	47/15	64 (45–84)/64 (51–77)	50/17	7	Hybrid	NR	NR	NR	43/8	23/11
Takeno	2013	Japan	91/166	77/147	63.7/64.2	NR	8	TA	NR	NR	NR	NR	NR
Kubo	2014	Japan	135/74	111/60	64.1 ± 8.2/62.2 ± 7.2	22/4	7	Hybrid	12/3	9/7	NR	112/41	23/33
Schneider	2014	UK	19/61	46/15	62.3 (35–74)/66.7 (45–79)	7/45	6	MIE	NR	NR	NR	16/36	2/24
Daiko	2015	Japan	31/33	28/28	66 (49–78)/65 (49–76)	NR	7	MIE	NR	NR	NR	23/32	8/1
Kauppi	2015	Finland	74/79	59/68	66 (51–85)/63 (39–82)	61/62	8	MIE	14/17	12/14	17/13	28/25	46/54
Law	1997	China	18/63	13/55	66 (43–80)/63 (36–84)	NR	7	MIE	NR	NR	NR	5/15	13/45
Chen	2010	China	67/38	45/25	61 ± 7/66 ± 6	NR	7	MIE	15/4	10/3	9/2	42/15	25/23
Gao	2011	China	96/78	89/70	58.5 ± 7.3/59.1 ± 6.4	NR	6	MIE	NR	NR	NR	54/40	42/38
Shen	2012	China	76/71	52/50	60.9 ± 9/62.6 ± 8.7	NR	6	MIE	NR	NR	NR	41/44	35/27
Liu	2012	China	98/105	67/71	62.3 ± 10.1/65.8 ± 7.6	NR	6	MIE	13/18	40/37	6/8	51/43	47/62
Mao	2012	China	34/38	28/26	62/60	NR	6	TA	NR	NR	NR	27/21	7/17
Wang	2012	China	260/322	194/232	61.6 ± 8.761.2 ± 8.8	37/44	6	MIE	NR	NR	NR	201/234	59/88
MU	2014	China	176/142	116/106	60 (55–66)/59 (54–62)	NR	6	MIE	NR	NR	NR	120/109	56/33
Meng	2014	China	94/89	65/63	59.7 ± 9.3/61.1 ± 6.7	NR	7	MIE	11/14	27/31	12/10	56/50	38/39
Zhang	2014	China	60/61	48/47	62.4 ± 8/61.8 ± 8.4	NR	6	MIE	NR	NR	NR	41/42	19/19
Chen	2015	China	59/59	42/40	57 (41–72)/56 (48–71)	NR	7	MIE	4/2	1/0	2/3	56/55	3/4
Yang	2015	China	62/62	45/45	62 ± 9/62 ± 8	NR	7	MIE	NR	NR	NR	44/43	18/19
Li	2015	China	89/318	66/227	73 (70–83)/73 (70–85)	NR	7	MIE	NR	NR	NR	64/188	25/126

*NT* neoadjuvant therapy, *NOS* Newcastle-Ottawa quality assessment scale, *MIO* minimally invasive oesophagectomy, including MIE,TA, and hybrid MIE, *OE* open esophagectomy, *MIE* total minimally invasive esophagectomy, *TA* thoracoscopic-assisted MIE, *Hybrid* hybrid minimally invasive oesophagectomy

### Preoperative clinical data

Fifty-seven studies reported patient’s age. There was no statistical significance between two groups after pooled analysis (WMD = −0.343, 95%CI = −1.200 ~ 0.514, *P*_*V*_ < 0.433). Thirty-three studies (5243 cases) reported that the patients in MIE group received more neoadjuvant therapy (Table 3, pooled OR = 1.364, 95% CI = 1.042 ~ 1.785, *P*_*V*_ = 0.024). Sixteen studies reported preoperative comorbidity, where there was no statistical significance between two groups (*P*_*V*_ > 0.05).

### Postoperative data

Forty-six studies (6260 cases) reported that operative time was higher in MIO group (Table 3, pooled WMD = 1.364, 95% CI = 10.912 ~ 37.943, *P*_*V*_ < 0.05). Forty studies (5285 cases) reported less blood loss in MIO group (WMD = −196, 95% CI = −255.195 ~ −136.926, *P*_*V*_ < 0.05). Duration of hospital stay (13,899 cases), including ICU stay (10,761 cases), were found to be significantly lower in MIO group (WMD = −1.599, 95% CI = (−2.680 ~ −0.518,*P*_*V*_ < 0.05 and WMD = −3.66, 95% CI = −4.891 ~ −2.428, *P*_*V*_ < 0.05). There was no significant difference between two groups in forty-six studies (6390 cases) reported for harvested lymph nodes (Table 3, WMD = −1.275, 95% CI = −5.851 ~ 3.301, *P*_*V*_ = 0.585). There was significant heterogeneity in the outcome among all the indices of postoperative data. Stratified analysis was performed according to ethnicity (Asian/Caucasian); however, heterogeneity still existed in subgroups. We then gradually removed small sample size, with emphasis on not altering the overall qualitative results.

### Complications

#### MIO and total complications

Thirty-five studies including 5991 cases reported total complications, where 41.5% (1206/2907) were allocated to MIE group and 48.2% (1486/3084) were allocated to OE group, with overall morbidity of 44.9% (2692/5991) (see Table 2).

**Table 2:** Outcomes of complication in included studies

Study	Total	Pulmonary	Circulatory system	Digestive system	AL	RLNP	STR	Mortality
MIO/OE	MIO/OE	MIO/OE	MIO/OE	MIO/OE	MIO/OE	MIO/OE	MIO/OE	MIO/OE
Nguyen	NR	2/6	1/1	1/2	3/4	0/4	0/1	0/1
Osugi	25/27	12/14	3/2	NR	2/1	11/9	4/4	NR
Kunisaki	NR	0/1	NR	NR	2/1	3/3	NR	NR
Bernabe	NR	NR	NR	7/8	NR	NR	NR	NR
Van den Broek	14/18	2/2	NR	3/5	2/3	2/3	2/4	NR
Braghetto	18/72	7/22	0/3	4/6	3/17	0/2	1/0	3/13
Bresadola	NR	1/2	1/0	NR	1/2	3/1	NR	NR
Shiraishi	NR	25/12	13/9	NR	12/9	42/10	NR	6/5
Smithers	207/76	106/44	60/24	83/9	17/11	8/0	25/14	7/3
Benzoni	NR	0/2	NR	0/1	1/1	1/1	NR	0/1
Fabian	15/31	1/18	5/8	1/0	3/3	1/2	0/3	1/4
Parameswaran	24/15	4/2	0/3	3/1	4/1	6/0	5/4	NR
Saha	3/6	NR	NR	NR	2/3	NR	NR	0/2
Zingg	19/20	17/33	NR	NR	11/11	NR	2/2	2/6
Pham	34/27	13/9	18/16	3/1	4/5	6/0	3/10	3/2
Perry	13/17	2/3	5/8	5/4	4/6	1/2	2/5	NR
Hamouda	NR	15/5	5/3	3/1	4/2	NR	3/0	NR
Safranek	NR	19/13	NR	17/4	11/1	10/1	5/5	3/1
Schoppmann	NR	5/17	NR	0/1	1/8	4/13	3/4	NR
Schröder	NR	NR	NR	NR	18/17	NR	NR	7/11
Mehran	NR	14/15	9/9	18/8	11/6	NR	NR	NR
Berger	31/32	10/22	1/6	NR	9/6	NR	NR	5/4
Lee	NR	11/20	NR	NR	10/18	NR	NR	4/8
Nafteux	44/61	17/47	11/13	13/6	5/10	NR	6/9	2/2
Yamasaki	26/38	7/15	3/6	0/2	6/4	17/20	3/5	0/2
Biere	NR	14/35	1/1	1/0	7/4	1/8	1/1	3/1
Maas	21/33	9/13	3/6	NR	4/3	3/5	2/5	0/1
Briez	50/83	22/60	NR	6/4	8/6	NR	NR	2/10
Kinjo	54/54	22/31	10/5	8/9	11/13	21/10	4/10	NR
Mamidanna	NR	276/1419	165/1035	NR	NR	NR	NR	46/274
Sihag	NR	1/33	5/19	NR	0/2	NR	3/5	0/2
Sundaram	28/41	5/19	9/19	26/10	4/4	1/1	10/11	2/1
Tsujimoto	13/16	2/10	NR	1/1	7/3	2/2	1/4	1/5
Javidfar	NR	9/26	29/56	19/33	5/7	3/0	22/38	3/7
Bailey	NR	15/18	4/9	1/0	1/0	NR	6/15	2/2
Ichikawa	94/117	20/33	17/38	4/5	14/27	60/77	2/2	0/8
Kitagawa	NR	6/14	NR	NR	NR	NR	13/20	2/1
Noble	NR	14/18	10/7	NR	5/2	NR	2/2	1/1
Parameswaran	42/12	7/2	2/1	14/2	NR	2/1	6/3	3/1
Takeno	39/69	NR	NR	NR	NR	NR	NR	4/15
Kubo	57/35	13/16	NR	2/0	10/7	37/14	18/19	2/2
Schneider	7/13	NR	NR	NR	NR	NR	NR	0/2
Daiko	10/12	NR	NR	NR	6/4	3/6	2/6	NR
Kauppi	37/48	13/15	17/27	5/14	5/5	0/4	12/11	NR
Law	NR	4/15	3/16	NR	0/2	4/8	NR	NR
Chen	NR	7/10	NR	2/0	1/0	NR	2/1	NR
Gao	31/36	13/11	NR	7/12	7/6	2/4	1/2	2/3
Shen	32/28	5/6	9/8	1/1	16/14	7/2	2/3	0/1
Liu	22/38	5/21	4/13	3/5	2/4	3/4	3/5	1/3
Mao	14/16	0/2	1/6	0/1	8/1	5/3	NR	NR
Wang	90/145	12/23	21/36	11/13	26/32	6/7	8/16	2/11
MU	28/22	6/4	NR	NR	12/4	NR	NR	1/1
Meng	24/41	9/24	4/11	2/2	6/7	4/4	3/4	1/4
Zhang	NR	4/7	3/5	3/2	3/2	2/1	2/7	NR
Chen	14/19	2/4	3/5	NR	2/3	4/5	1/1	NR
Yang	19/31	NR	NR	NR	NR	NR	NR	NR
Li	32/137	8/51	9/34	2/5	19/45	18/49	4/19	3/16

*AL* anastomotic leak, *RLNP* recurrent laryngeal nerve palsy, *STR* surgical technology-related, *Mortality* in-hospital/30-day mortality

**Fig. 2:**
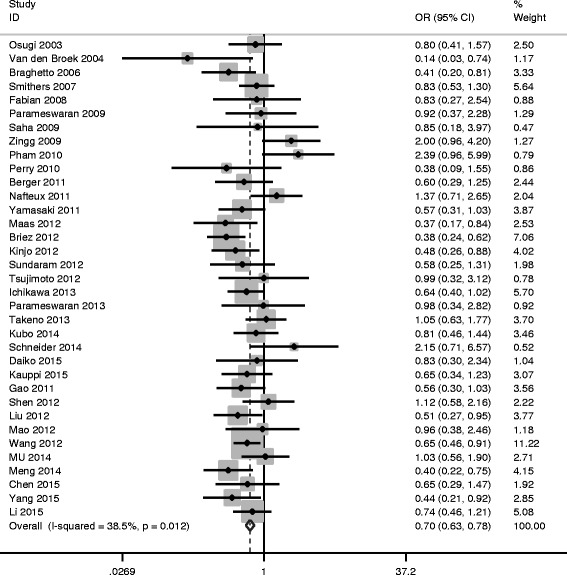
Meta-analysis for MIE and total complications

Low heterogeneity was found among studies (*I* ^2^ = 38.5%, *P*_*Q*_ = 0.012), so the fixed effects model was used (see Table 3). The pooled OR = 0.70, 95% CI = 0.626 ~ 0.781, *P*_*V*_ < 0.05 indicated total complication was significantly lower in MIO group (Fig. 2). Publication bias was assessed by Egger’s Test and Begg’s Funnel Plot; no publication bias could be discovered (*P*_*E*_ = 0.178).

**Table 3:** Differences between MIO and OE surgery patients

Variables	No. studies	WMD/OR (95%CI)	*P*_*V*_	*P*_*Q*_	*I* ^2^ (%)	*P*_*E*_
Age, years	57 (*n* = 15790)	−0.343 (−1.200, 0.514)	0.433	<0.05	68.1	0.059
NT	34 (*n* = 5138)	1.364 (1.042,1.785)	0.024	<0.05	73.0	0.362
Comorbidity						
Cardiovascular	16 (*n* = 10337)	0.913 (0.815,1.022)	0.112	0.030	44.2	0.930
Pulmonary	15 (*n* = 9779)	0.949 (0.819,1.099)	0.485	0.881	0	0.722
Diabetes	15 (*n* = 9983)	0.942 (0.798,1.111)	0.476	0.457	0	0.082
Operating time, min	46 (*n* = 6260)	24.427 (10.912,37.943)	<0.05	<0.05	96.1	0.155
Blood loss, ml	40 (*n* = 5285)	−196.060 (−255.195,-136.926)	<0.05	<0.05	98.9	0.592
LN harvest	46 (*n* = 6390)	−1.275 (−5.851,3.301)	0.585	<0.05	99.8	0.786
LOS, day	45 (*n* = 13899)	−3.660 (−4.891,-2.428)	<0.05	<0.05	86.0	0.175
ICU stay, day	27 (*n* = 10761)	−1.599 (−2.680, −0.518)	0.004	<0.05	98.2	0.078
Complication						
Total complication	35 (*n* = 5991)	0.700 (0.626,0.781)	<0.05	0.012	38.5	0.178
Pulmonary	50 (*n* = 14781)	0.527 (0.431, 0.645)	<0.05	<0.05	60.3	<0.05
Circulatory system	36 (*n* = 12883)	0.770 (0.681,0.872)	<0.05	0.427	2.4	0.386
Digestive system	21 (*n* = 4081)	1.097 (0.835,1.442)	0.507	0.083	31.7	0.664
AL	50 (*n* = 7528)	1.023 (0.870,1.202)	0.785	0.304	8.5	0.018
RLNP	37 (*n* = 5429)	1.108 (0.917,1.339)	0.289	0.089	24.8	0.014
STR	39 (*n* = 5991)	0.639 (0.522,0.781)	<0.05	0.918	0	0.206
Mortality	38 (*n* = 14132)	0.668 (0.539,0.827)	<0.05	0.944	0	0.508

*NT* neoadjuvant therapy, *LN* lymph node, *LOS* length of hospital stay, *ICU* intensive care unit, *AL* anastomotic leak, *RLNP* recurrent laryngeal nerve palsy, *STR* surgical technology-related, *Mortality* in-hospital/30-day mortality, *P*_*V*_ the *P* value for pooled, *P*_*Q*_ the *P* value for *Q* test, *P*_*E*_ the *P* value for Egger’s test

#### MIO and pulmonary complications

Fifty studies including 14,781 cases reported pulmonary complications, where 17.1% (813/4761) were in MIO group and 22.6% (2264/10,020) were in OE group, with overall morbidity of 20.8% (3077/14,781).

**Fig. 3:**
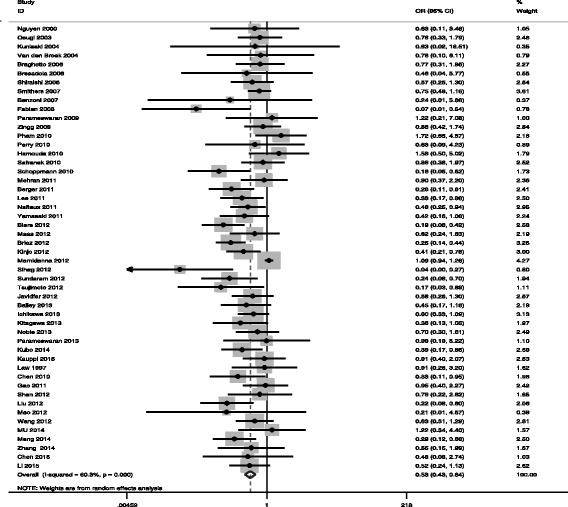
Meta-analysis for MIE and pulmonary complications

**Fig. 4:**
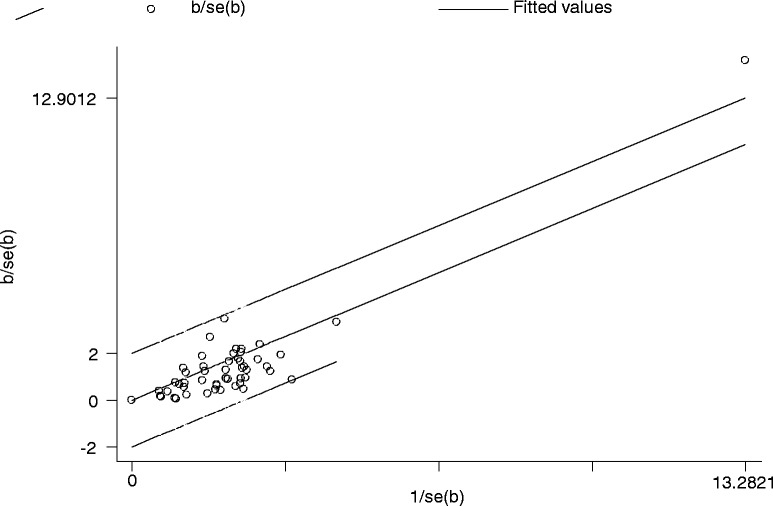
Galbraith plot of MIE and pulmonary complications

**Fig. 5:**
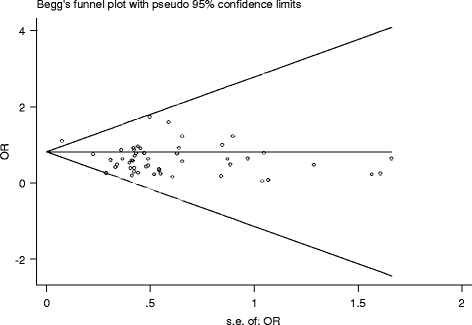
Begg’s Test of MIE and pulmonary complications

There was very strong evidence of reduced risk of pulmonary complications in the MIO group (OR = 0.527, 95%CI = 0.431 ~ 0.645, *P*_*V*_ < 0.05), with statistical heterogeneity (*I* ^2^ of 60.3%, *P*_*Q*_ = 0.012) (Fig. 3, Table 3). In order to find out other sources of heterogeneity, Galbraith Plot Analysis was performed to identify which study results in the heterogeneity (Fig. 4). Pham et al. [52] and Mamidanna et al. [66] were outliers from the Galbraith Plot Analysis and *I* ^2^ values decreased after removing the study (OR = 0.502 95% CI = 0.425 ~ 0.592, *P*_*V*_ < 0.05, *I* ^2^ = 26.6%, *P*_*Q*_ = 0.05). However, the funnel plot figure (Fig. 5) showed significant statistical difference (*P*_*E*_ < 0.05), indicating the possibility of publication bias.

#### MIO and mortality

**Fig. 6:**
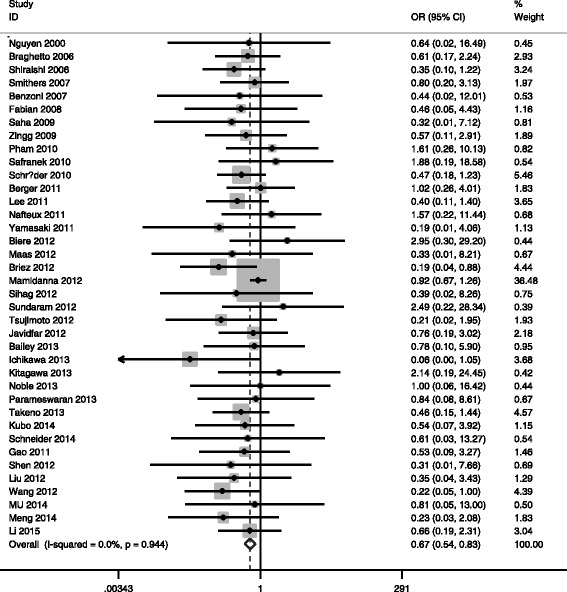
Meta-analysis for MIE and Mortality

Thirty-eight studies addressed the mortality (MIO 4379 vs OE 9753). The mortality risk was 3.8% (124/4379) in MIO group versus 4.5% (437/9753) in OE group. There was very strong evidence of reduced mortality in MIO group (OR = 0.668, 95% CI = 0.539 ~ 0.827, *P*_*V*_ < 0.05), with statistical homogeneity (*I* ^2^ of 0%, *P*_*Q*_ = 0.944) (Fig. 6).

#### MIO and cardiovascular complications

**Fig. 7:**
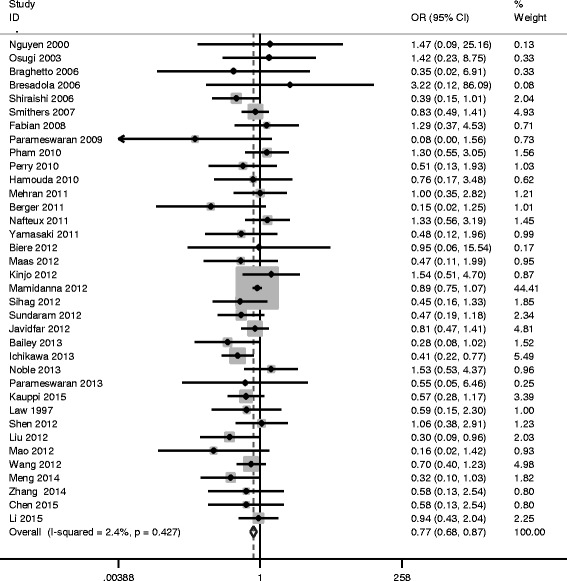
Meta-analysis of MIE and cardiovascular complications

Thirty-six studies reported cardiovascular complications (MIO 3745 vs OE 9138). There was very strong evidence of reduced cardiovascular complications in MIO group (OR = 0.770, 95% CI = 0.681 ~ 0.872, *P*_*V*_ < 0.05), with statistical homogeneity (*I* ^2^ of 2.4%, *P*_*Q*_ = 0.427) (Fig. 7).

#### MIO and surgical technology related (STR) complications

**Fig. 8:**
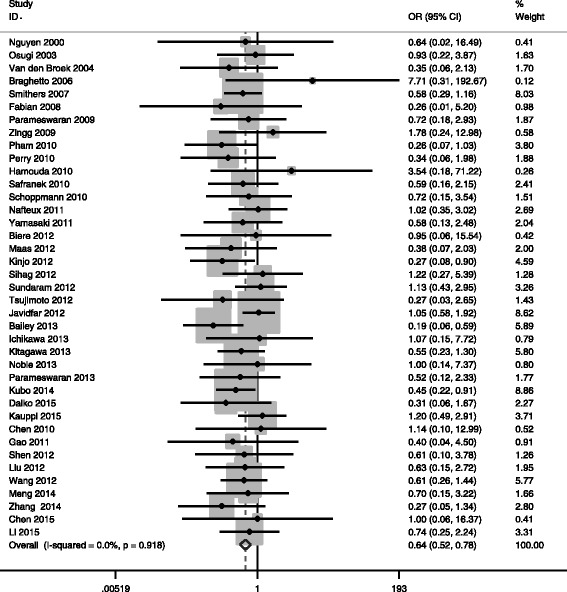
Meta-analysis of MIE and STR complications

Thirty-nine studies reported STR complications (MIO2933 vs OE 3058). There was very strong evidence of reduced STR complications in MIO group (OR = 0.770, 95% CI = 0.681 ~ 0.872, *P*_*V*_ < 0.05), with statistical homogeneity (*I* ^2^ of 2.4%, *P*_*Q*_ = 0.918) (Fig. 8 and Table 3).

#### MIO and gastrointestinal complications

Twenty-one studies reported gastrointestinal complications (MIO 1872 vs OE 2209). There was no evidence of reduced gastrointestinal complications in MIO group (OR = 1.097, 95% CI = 0.835 ~ 1.442, *P*_*V*_ = 0.507), with statistical homogeneity (*I* ^2^ of 31.7%, *P*_*Q*_ = 0.083) (Table 3).

#### MIO and anastomotic leak (AL)

Fifty studies reported anastomotic leak (MIO 3680 vs OE 3848). There was no evidence of reduced anastomotic leak in MIO group (OR = 1.023, 95% CI = 0.870 ~ 1.202, *P*_*V*_ = 0.785), with statistical homogeneity (*I* ^2^ of 8.5%, *P*_Q_ = 0.304) (Table 3).

#### MIO and recurrent laryngeal nerve palsy (RLNP)

Thirty-seven studies reported recurrent laryngeal nerve palsy (MIO 2624 vs OE 2805). There was no evidence of reduced RLNP in MIO group (OR = 1.108, 95% CI = 0.917 ~ 1.339, *P*_*V*_ = 0.289), with statistical homogeneity (*I* ^2^ of 24.8%, *P*_Q_ = 0.089) (Table 3).

#### Publication bias analysis

**Fig. 9:**
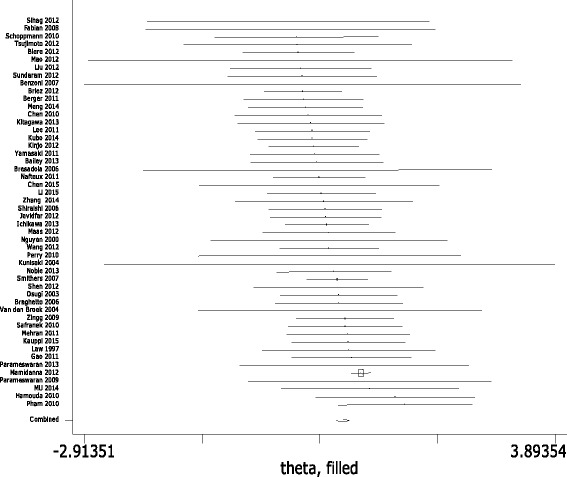
The “trim and fill” method for MIE and pulmonary complications

Publication bias was assessed by Egger’s Test and Begg’s Funnel Plot. Begg’s Funnel Plot is shown in Fig. 5, with significant statistical difference (*P*_*E*_ < 0.05) (Table 3). This indicated the possibility of publication bias, so sensitivity analysis using “trim and fill” method was carried out, with the aim to impute hypothetically negative unpublished studies, to mirror the positive studies that cause funnel plot asymmetry [35], and to show consistent and stable results between MIO and pulmonary complications (Fig. 9), anastomotic leak, and recurrent laryngeal nerve palsy.

#### Sensitivity analysis

**Fig. 10:**
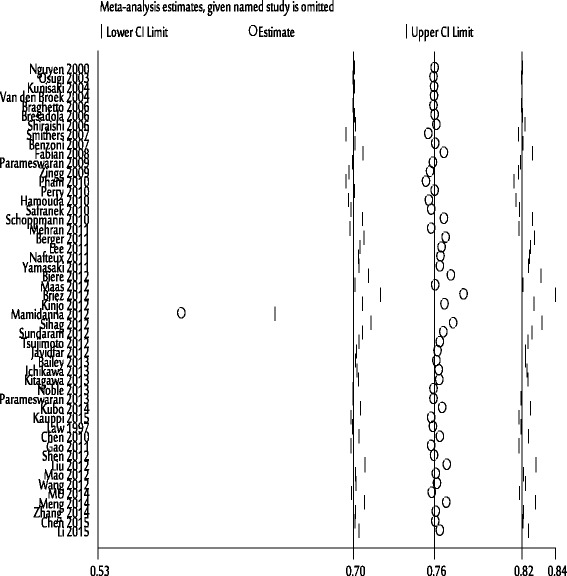
The sensitivity analysis of MIE and pulmonary complications

As sample size for cases and controls in all studies is not same (ranging from 9 to 6347), we gradually removed small sample size without altering the qualitative overall results. According to the sensitivity analysis shown in Fig. 10, we removed the Mamidanna et al. [66], without alteration, where *I* ^2^ values decreased, indicating that the results were stable.

## Discussion

MIO has been investigated for decades and is considered to be advantageous compared to OE. However, in the previous studies, the analyzed groups of patients who underwent MIO were small and the reports were mostly retrospective comparative studies, and there was no consensus as to which operative method is superior [94]. Therefore, an updated meta-analysis is performed, which includes the largest and the most complete collections of published data.

We found higher operative duration in the MIO group, consistent with Kunisaki’s [40], Shiraishi’s [45], and randomized controlled trials [30] reported, perhaps due to surgeons’ familiarization with a new and complex techniques. Blood loss in the MIO group was found to be lower compared with OE, in accordance with the results of several case reported and recently published meta-analyses [14, 20].

A shorter hospital stay in the MIO group indicated a faster postoperative recovery than OE group, consistent with other published meta-analyses [14, 20, 21, 30].

We did not find a significant number of harvested lymph nodes in the MIO group [23]. However, significant heterogeneity was seen among all indices of postoperative data, explained by the fact that postoperative data are dependent on operator and tumor characteristics.

Total complication rates varied between 20.5 and 63.5% (Table 2). The MIO group showed lower total complication rates, pulmonary complications occupying the major part. However, a number of studies have reported significantly lower pulmonary complications for those who underwent MIO 17.1% (813/4761) versus OE 22.6% (2264/10,020), with overall morbidity of 20.8% (3077/14,781), consistent with the result of 3.1–37.0% from other studies [15–20, 45, 58–76, 95].

Kinugasa et al. and Ferguson et al. [95, 96] noted that development of pneumonia post procedure was associated with worse prognosis for overall survival (*P* < 0.01). In addition, Dumont et al. [97] also showed that two thirds of all fatal complications were respiratory in nature. Sauvanet et al. [98] reported that pulmonary morbidity was associated with age >60, with no significant differences in two groups.

The pooled OR of 0.527 showed MIO to be more advantageous than OE in reducing pulmonary morbidity. Although statistical heterogeneity and publication bias were found, we demonstrated the superiority of MIO through statistical methods. However, several factors have been associated with pulmonary complications post procedure, including preoperative status, intraoperative details, and postoperative details [99].

Gex et al. reported that overall 30-day mortality rate was 4.3% between 2004 and 2009, compared with 7.6% in 2002 and 2003, and 11.7% in 1997 and 1999 [100]. Our study found the overall 30-day mortality rate of 5.8% and the pooled OR of 0.668, showing that MIO to be advantageous than OE in reducing mortality. The main advantages of MIO over conventional OE are minimal trauma, small incision, less blood loss, etc. [6]. Other factors independently associated with 30-day mortality included TNM staging, preoperative neoadjuvant therapy, comorbidity, diabetes, increased age, and intraoperative blood loss. However, there was no difference between two groups in terms of age and comorbidity. We found increased number of patients having neoadjuvant therapy in MIO group and patients selected for MIO were always in the early stages. The bias in the selection of patients may have influenced the accuracy of the conclusion, which should be taken into consideration.

Arrhythmia, heart failure, pulmonary embolism, and other cardiovascular complications are recognized as common problems that caused significant morbidity and mortality. Zhou et al. [24] reported significant decrease in the morbidity of arrhythmia and pulmonary embolism in MIO group. Corresponding to this, (see Table 3), we found MIO to be superior to OE in reducing morbidity of system complications, according to the pooled OR = 0.777. Weidenhagent et al. [101] also indicated that the perforation from minimally invasive surgery as such could decrease the risks leading to arrhythmia.

Rizk et al. [102] indicated that “surgical technology related complications,” defined as complications caused directly by operative techniques, had no relationship with overall survival post procedure. However, in our meta-analysis, we found strong evidence of reduced risk of STR complications in the MIO group.

Anastomotic leakage (AL) is a serious complication of esophageal resection and is associated with significant morbidity and mortality [4]. In accordance with Zhou et al’s conclusion [17], we also did not find the evidence of reduced risk of anastomotic leak in the MIO group. Similarly, we also did not find any significant differences in two groups in terms of RLNP and gastrointestinal complications.

Although we conducted comprehensive meta-analysis, our study still has its limitations. (1) Out of 57 studies, only one study is randomized controlled trial (RCT), while others were case-control or cross-sectional designs. Seven studies were of small sample size, which might have influenced the final results of our study. (2) Patients selected for MIO are unlikely to have been representative of the general population of esophageal cancer. We found more patients having neoadjuvant therapy in MIO group, and the patients selected for MIO were always in the early stages, creating selection bias. (3) In order to highlight the advantages of MIO, surgeons would prefer to publish positive results, and unsatisfactory results may have been less inclined in their papers; all these can lead to publication bias. (4) In our study, we compared MIO with OE. MIO consists of different procedures. Although we performed a subgroup analysis according to different procedures, the results were also not qualitatively altered. However, lots of differences exist among these procedures, which will affect the quality of this meta-analysis, and the learning curve of MIO is quite steep, which may influence the outcome of MIE. These limitations may result in an overestimation or underestimation of the effect of MIO.

In addition, 19 studies did the follow-up visit, and all those studies indicated that the 3-year survival, 5-year survival, and overall recurrence rate did not differ between the two groups. Due to the difficulty in data extraction, no pooled analysis was performed, which may have influential role in this study.

## Conclusions

In summary, this meta-analysis indicates that MIO is a feasible and a reliable surgical procedure and is superior to OE, with less perioperative complications and in-hospital mortality. However, due to certain limitations of this study, as aforementioned above, further large sample and RCT studies are needed to estimate the effect of MIO and establish the guidelines for future.

## Abbreviations

MIE: Minimally invasive esophagectomy
MIO: Minimally invasive oesophagectomy
OE: Open esophagectomy
RLNP: Recurrent laryngeal nerve palsy
STR: Surgical technology related
TA: Thoracoscopic assisted
